# Author Correction: Amyloid modifier SERF1a interacts with polyQ-expanded huntingtin-exon 1 via helical interactions and exacerbates polyQ-induced toxicity

**DOI:** 10.1038/s42003-023-05389-7

**Published:** 2023-10-09

**Authors:** Tien-Ying Tsai, Chun-Yu Chen, Tien-Wei Lin, Tien-Chang Lin, Feng-Lan Chiu, Orion Shih, Ming-Yun Chang, Yu-Chun Lin, An-Chung Su, Chiung-Mei Chen, U-Ser Jeng, Hung-Chih Kuo, Chi-Fon Chang, Yun-Ru Chen

**Affiliations:** 1 Genomics Research Center, Academia Sinica, 128, Academia Rd., Sec. 2, Nankang District, Taipei, 115 Taiwan; 2 Chemical Biology and Molecular Biophysics Program, Taiwan International Graduate Program, Institute of Biological Chemistry, Academia Sinica, 128, Academia Road, Sec. 2. Nankang, Taipei, 115 Taiwan; 3 Institute of Biochemical Sciences, National Taiwan University, Taipei, Taiwan; 4 National Synchrotron Radiation Research Center, Hsinchu, 300 Taiwan; 5 Department of Chemical Engineering, National Tsing Hua University, Hsinchu, 300 Taiwan; 6 Institute of Cellular and Organismic Biology, Academia Sinica, 128, Academia Rd., Sec. 2, Nankang District, Taipei, 115 Taiwan; 7 Taiwan International Graduate Program in Interdisciplinary Neuroscience, National Yang Ming Chiao Tung University and Academia Sinica, Taipei, Taiwan; 8 Department of Neurology, Linkou Chang Gung Memorial Hospital and College of Medicine, Chang Gung University, Taoyuan, 333 Taiwan

**Keywords:** Protein aggregation, Huntington's disease

Correction to: *Communications Biology* 10.1038/s42003-023-05142-0, published online 21 July 2023.

In the original version of the article, Ming-Yun Chang was missing from the author list. This has now been corrected and the full author list is:

Tien-Ying Tsai^1,2,3§^, Chun-Yu Chen^1§^, Tien-Wei Lin^1^, Tien-Chang Lin^4,5^, Feng-Lan Chiu^6^, Orion Shih^4^, Ming-Yun Chang^1,7^, Yu-Chun Lin^1,^ An-Chung Su^5^, Chiung-Mei Chen^8^, U-Ser Jeng^4,5^, Hung-Chih Kuo^6^, Chi-Fon Chang^1^, and Yun-Ru Chen^1*^

The affiliation of Ming-Yun Chang has also been added:

^7^ Taiwan International Graduate Program in Interdisciplinary Neuroscience, National Yang Ming Chiao Tung University and Academia Sinica, Taipei, Taiwan

The “Author contributions” section has been modified to read:

T.Y.T. performed the ITC and ELISA experiments, wrote the manuscript, and revised the manuscript. C.Y.C. performed most experiments and wrote the first version of the manuscript. T.W.L. and M.Y.C performed ICC of iPSC-derived neurons. F.L.C. and H.C.K. provided iPSC-derived neurons. T.C.L., O.S., A.C.S., and U.S.J. performed SAXS experiments, analyzed the data, and wrote the SAXS part of the manuscript. Y.C.L. performed mouse qPCR experiment. C.M.C. provided human plasma. C.F.C. contributed to NMR data analysis and wrote the method for NMR. Y.R.C. conducted the research and edited the manuscript.

