# Author Correction: Amyloid modifier SERF1a interacts with polyQ-expanded huntingtin-exon 1 via helical interactions and exacerbates polyQ-induced toxicity

**DOI:** 10.1038/s42003-024-06302-6

**Published:** 2024-05-18

**Authors:** Tien-Ying Tsai, Chun-Yu Chen, Tien-Wei Lin, Tien-Chang Lin, Feng-Lan Chiu, Orion Shih, Ming-Yun Chang, Yu-Chun Lin, An-Chung Su, Chiung-Mei Chen, U-Ser Jeng, Hung-Chih Kuo, Chi-Fon Chang, Yun-Ru Chen

**Affiliations:** 1 Genomics Research Center, Academia Sinica, 128, Academia Rd., Sec. 2, Nankang District, Taipei, 115 Taiwan; 2 Chemical Biology and Molecular Biophysics Program, Taiwan International Graduate Program, Institute of Biological Chemistry, Academia Sinica, 128, Academia Road, Sec. 2. Nankang, Taipei, 115 Taiwan; 3 Institute of Biochemical Sciences, National Taiwan University, Taipei, Taiwan; 4 National Synchrotron Radiation Research Center, Hsinchu, 300 Taiwan; 5 Department of Chemical Engineering, National Tsing Hua University, Hsinchu, 300 Taiwan; 6 Institute of Cellular and Organismic Biology, Academia Sinica, 128, Academia Rd., Sec. 2, Nankang District, Taipei, 115 Taiwan; 7 Taiwan International Graduate Program in Interdisciplinary Neuroscience, National Yang Ming Chiao Tung University and Academia Sinica, Taipei, Taiwan; 8 Department of Neurology, Linkou Chang Gung Memorial Hospital and College of Medicine, Chang Gung University, Taoyuan, 333 Taiwan

**Keywords:** Protein aggregation, Huntington's disease

Correction to: *Communications Biology* 10.1038/s42003-023-05142-0, published online 21 July 2023

The original version of this Article contained a repetition of two images in Fig. 1E, in which the ellipticity plots for TrxHttex-1 (29Q) with and without SERF1a were identical. The correct version of Fig. 1E is:
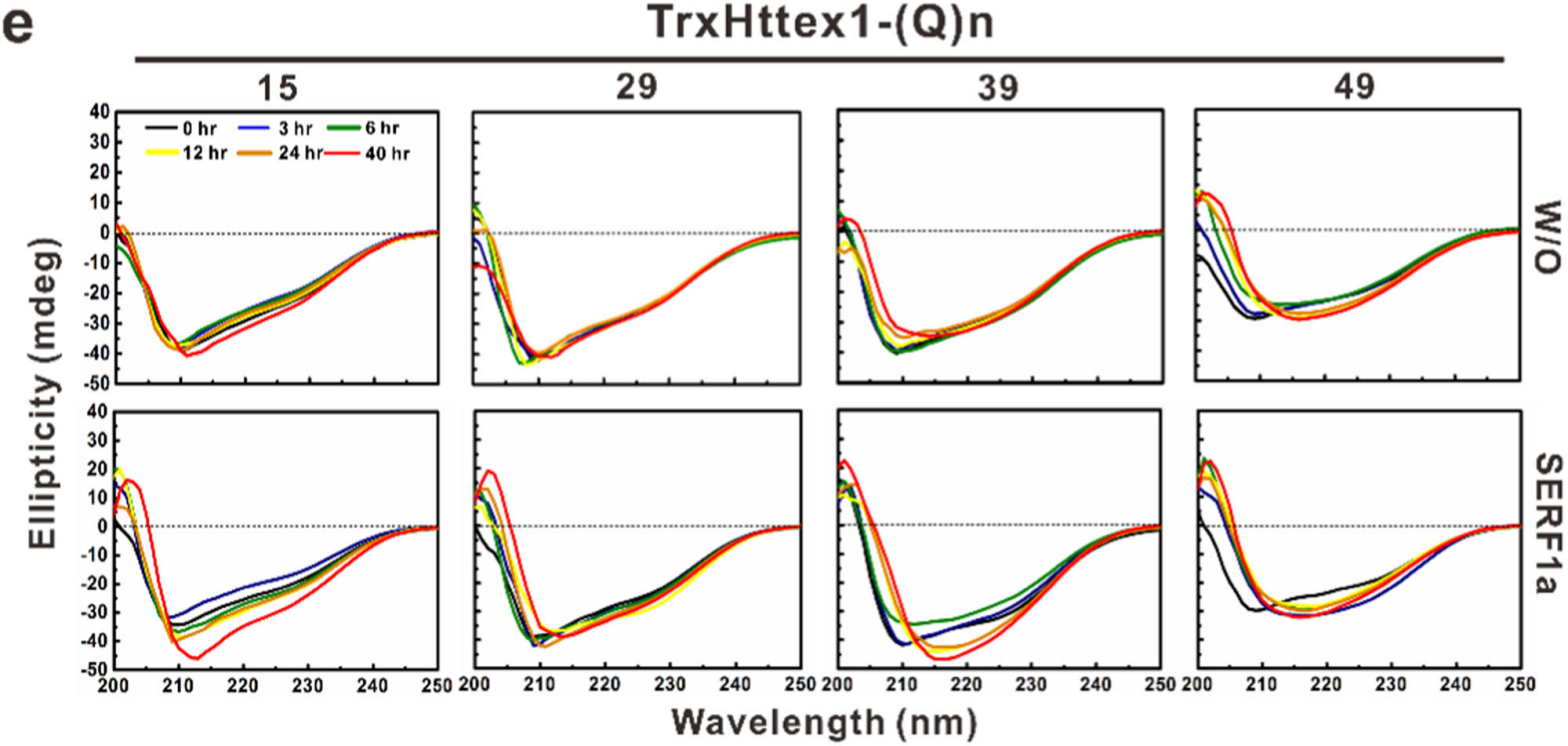


This has now been corrected in both the PDF and HTML versions of the Article.

