# Supplementary material for: Amyloid modifier SERF1a interacts with polyQ-expanded huntingtin-exon 1 via helical interactions and exacerbates polyQ-induced toxicity
Source: Commun Biol. 2023 Jul 21;6:767. doi: 10.1038/s42003-023-05142-0 (PMC10361993; doi:10.1038/s42003-023-05142-0)
Supplement: Supplementary file 2 — Supplementary Information [file 42003_2023_5142_MOESM2_ESM.pdf]

Supplementary Information

Amyloid Modifier SERF1a Interacts with PolyQ-expanded Huntingtin-exon 1 via Helical Interactions and Exacerbates PolyQ-induced Toxicity

Supplementary Figure

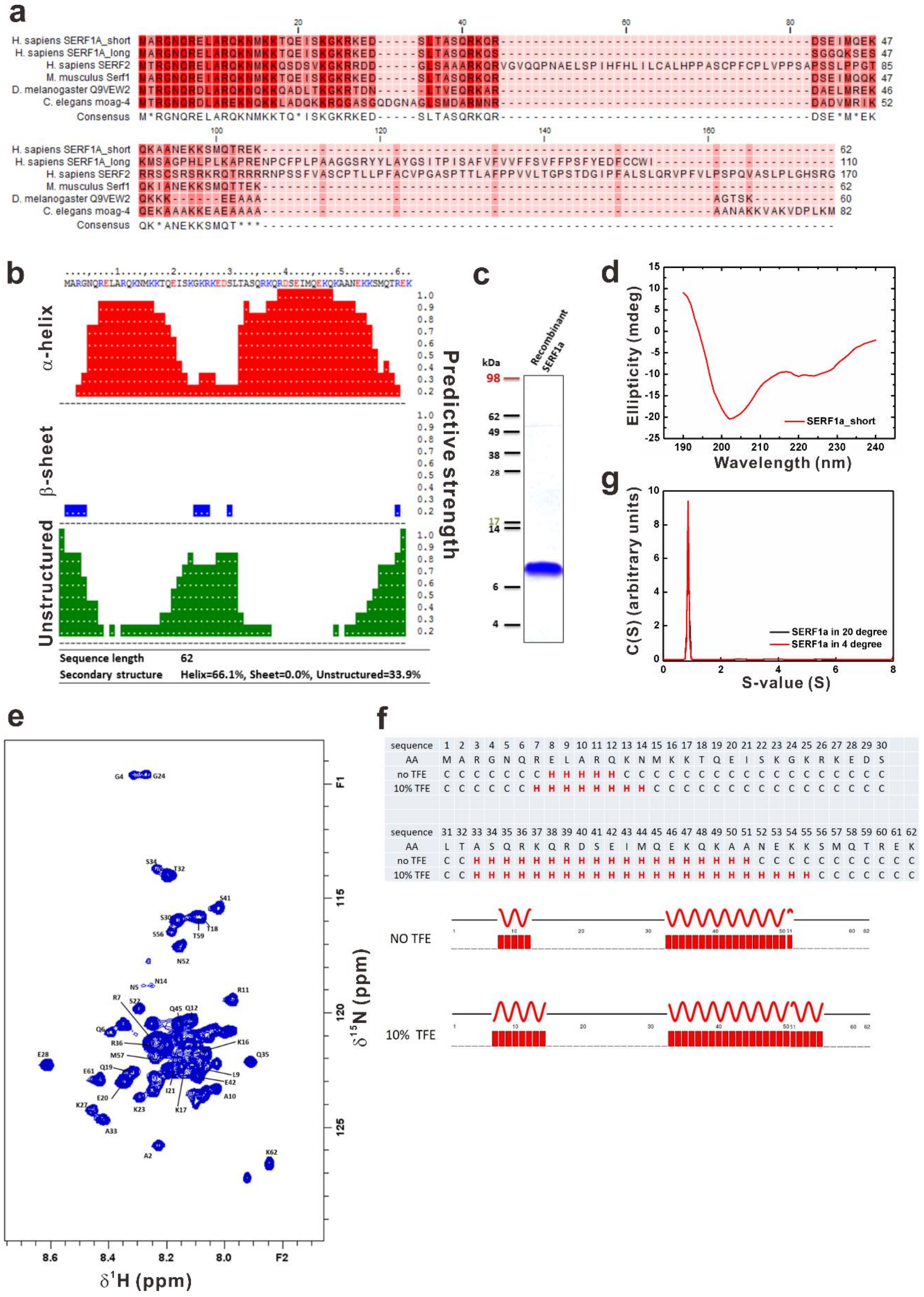

Supplementary Figure 1. SERF1a protein sequence alignment and secondary structure analysis. (a) Alignment of human SERF1a proteins with representative orthologs. The conservation level of residues from low to high were highlighted in red, from light to dark (CLC Sequence Viewer 8.0). **(b)** Secondary structure prediction of short-form SERF1a by PHDsec algorithm. The structure of α-helix, β-sheet, and unstructured regions of SERF1a were predicted. The prediction showed SERF1a adopts a helix-loop-helix protein containing 66% α-helices and 34% loop. The acidic residues were colored in red, and the basic residues were colored in blue. **(c)** Purified recombinant SERF1a protein in SDS-PAGE. **(d)** Far-UV CD spectroscopy of SERF1a. **(e)** HSQC spectrum of ^{15}N-labeled SERF1a. **(f)** Secondary structure of SERF1a with and without 10% TFE, determined by Chemical Shift Index 3.0 based on NMR backbone assignment of SERF1a. **(g)** Sedimentation velocity analysis of SERF1a at 4 °C or 20 °C. Data were analyzed using a continuous c(s) distribution model implemented in the program SEDFIT. The result showed SERF1a exists as a monomeric species.

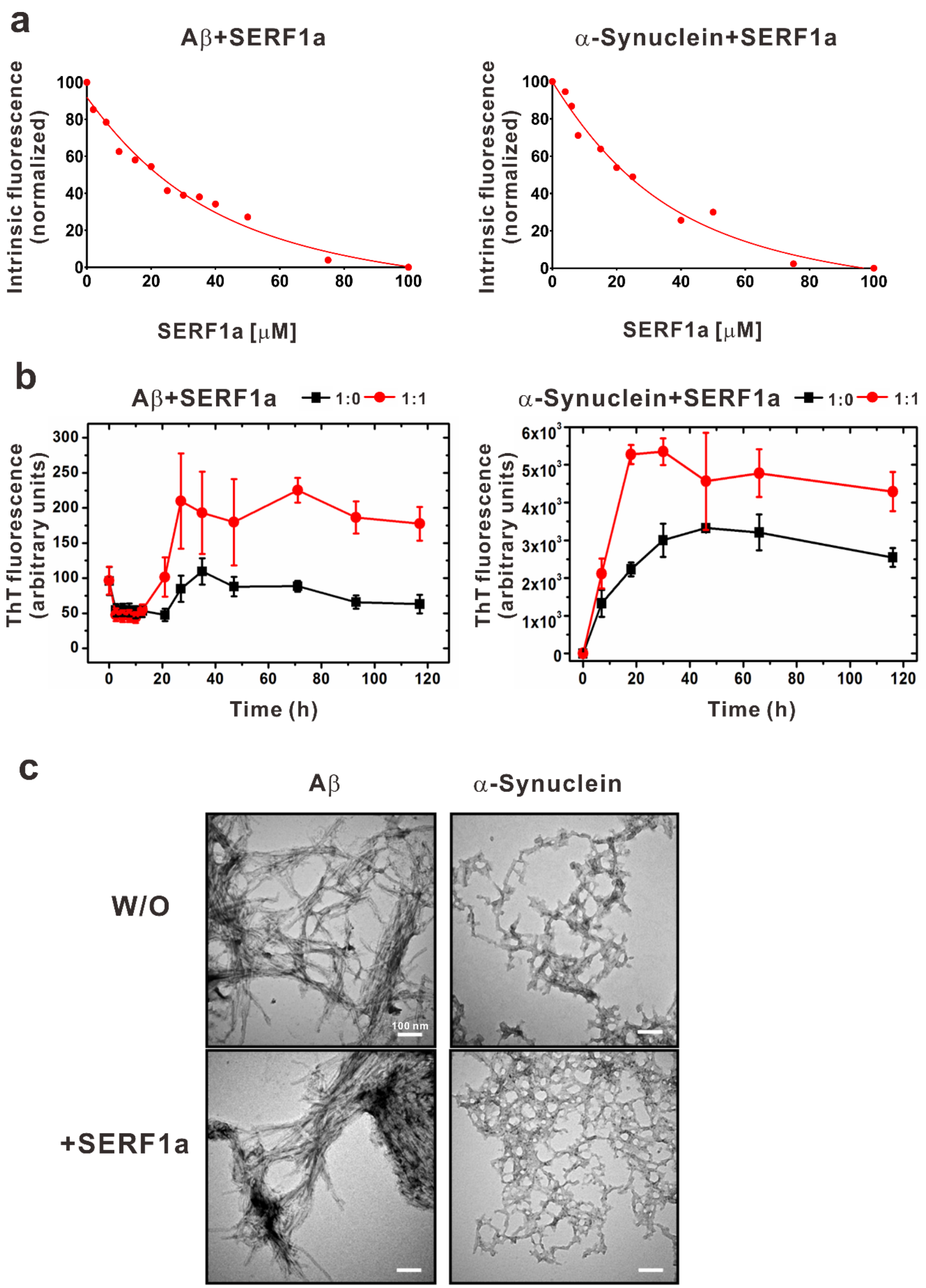

Supplementary Figure 2. SERF1a interacts with Aβ and α-synuclein and enhances their fibril formation. (a) Fluorescence titration of Aβ or α-synuclein with SERF1a. The

intrinsic fluorescence of Aβ and α-synuclein was monitored upon titration of SERF1a. Quenching of fluorescence showed SERF1a interacts with Aβ or α-synuclein protein. **(b)** ThT assay of Aβ (25 μM) and α-synuclein (50 μM) with and without SERF1a. The samples were incubated at 25 °C with continuous shaking at 800 rpm. ThT fluorescence at 485 nm was monitored with excitation at 442 nm. The error bars represent standard deviation. **(c)** TEM images of endpoint products after incubation for 118 hr. In the presence of SERF1a, the fibrils were more crowed and bundled (scale bar, 100 nm).

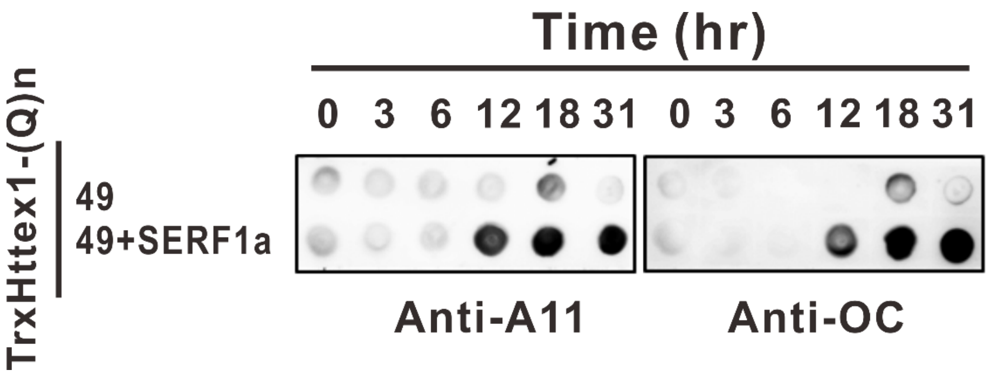

Supplementary Figure 3. Dot-blot analysis of TrxHttex1-49Q with and without SERF1a at different incubation times and probed by conformational amyloid antibodies. Time-course samples of 50 μM TrxHttex1-49Q with and without equimolar SERF1a were dotted and probed by conformational specific A11 and OC antibodies. In the presence of SERF1a, TrxHttex1-49Q showed faster appearance of A11 and OC signals.

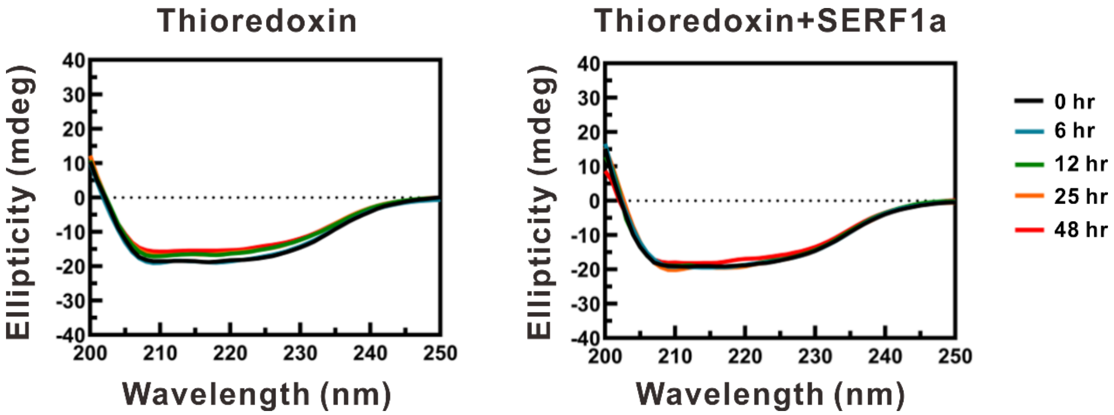

Supplementary Figure 4. Far-UV CD analysis of thioredoxin with and without SERF1a at different incubation times. Buffer background was subtracted from the spectrum of thioredoxin alone, and SERF1a alone spectrum was subtracted from that of thioredoxin with SERF1a.

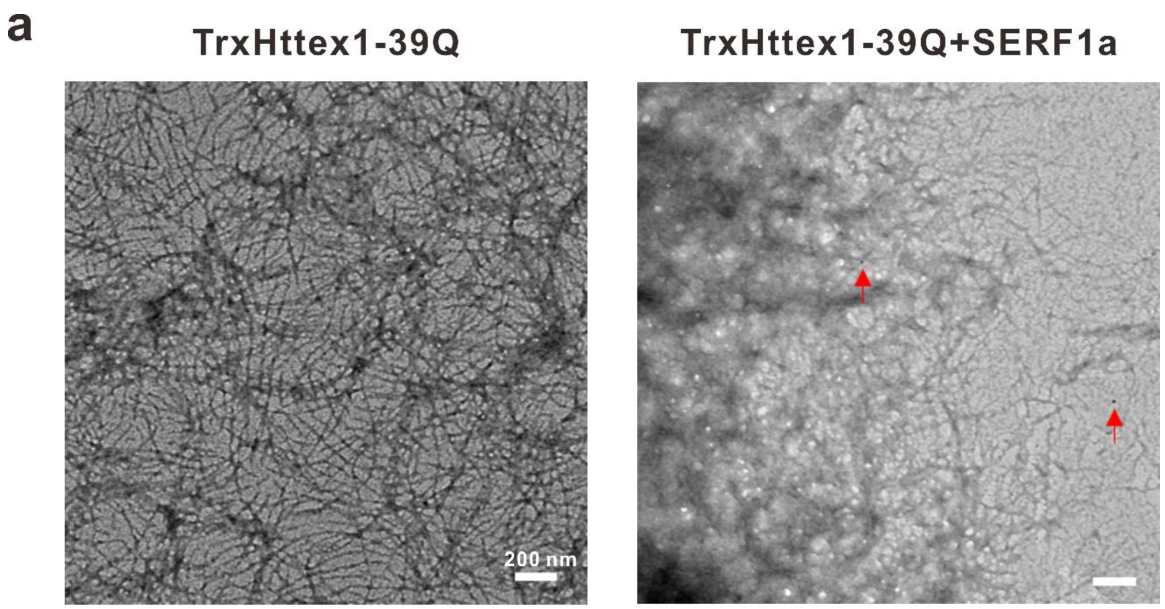

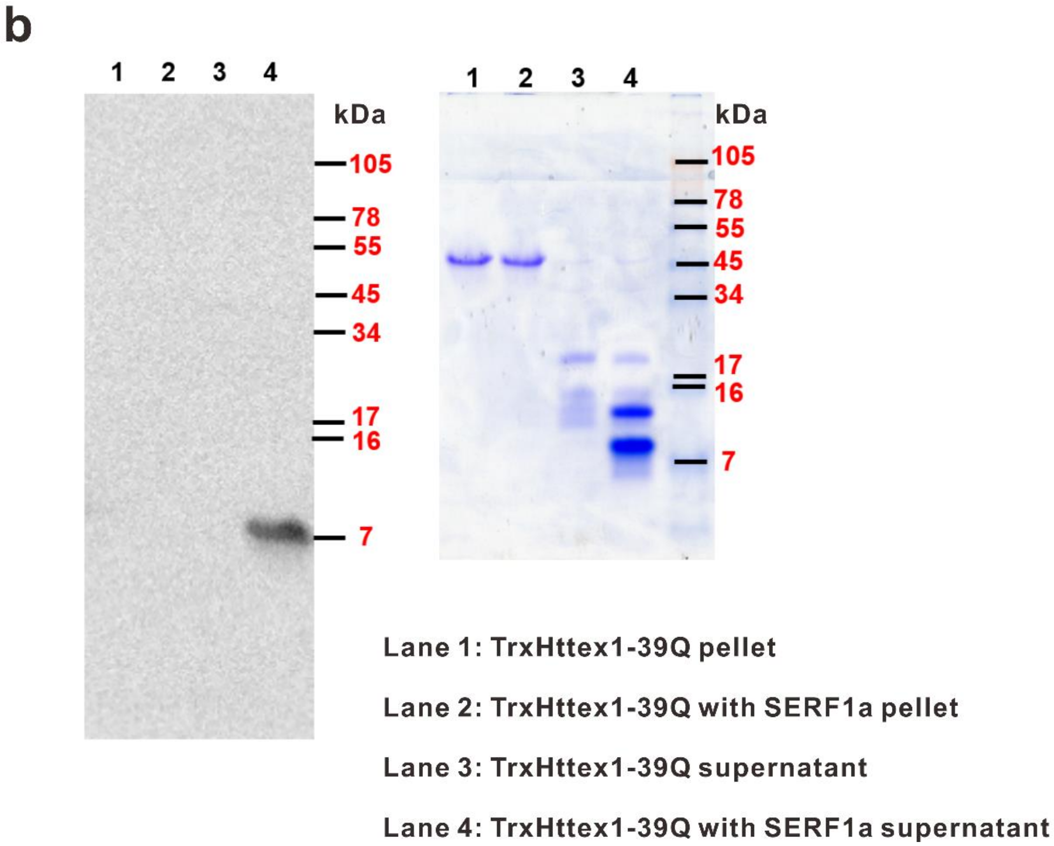

Supplementary Figure 5. SERF1a dissociates from Httex1 fibrils. (a) TEM images of the end-point products of 50 μM TrxHttex1-39Q with and without equimolar SERF1a

immunogold staining. The 10 nm golds were marked by red arrows. The scale bars are 200 nm. (b) Western blot and SDS gel of the end-point products of 50 μM TrxHttex1-39Q with and without equimolar SERF1a incubation with 300 rpm shaking at 37 °C for at least 40 hr. The samples were centrifuged to separate the supernatant and the pellet, and the latter was washed by PBS for three times. Lane 1: pellet of TrxHttex1-39Q; Lane 2: pellet of TrxHttex1-39Q with SERF1a; Lane 3: supernatant of TrxHttex1-39Q; Lane 4: supernatant of TrxHttex1-39Q with SERF1a.

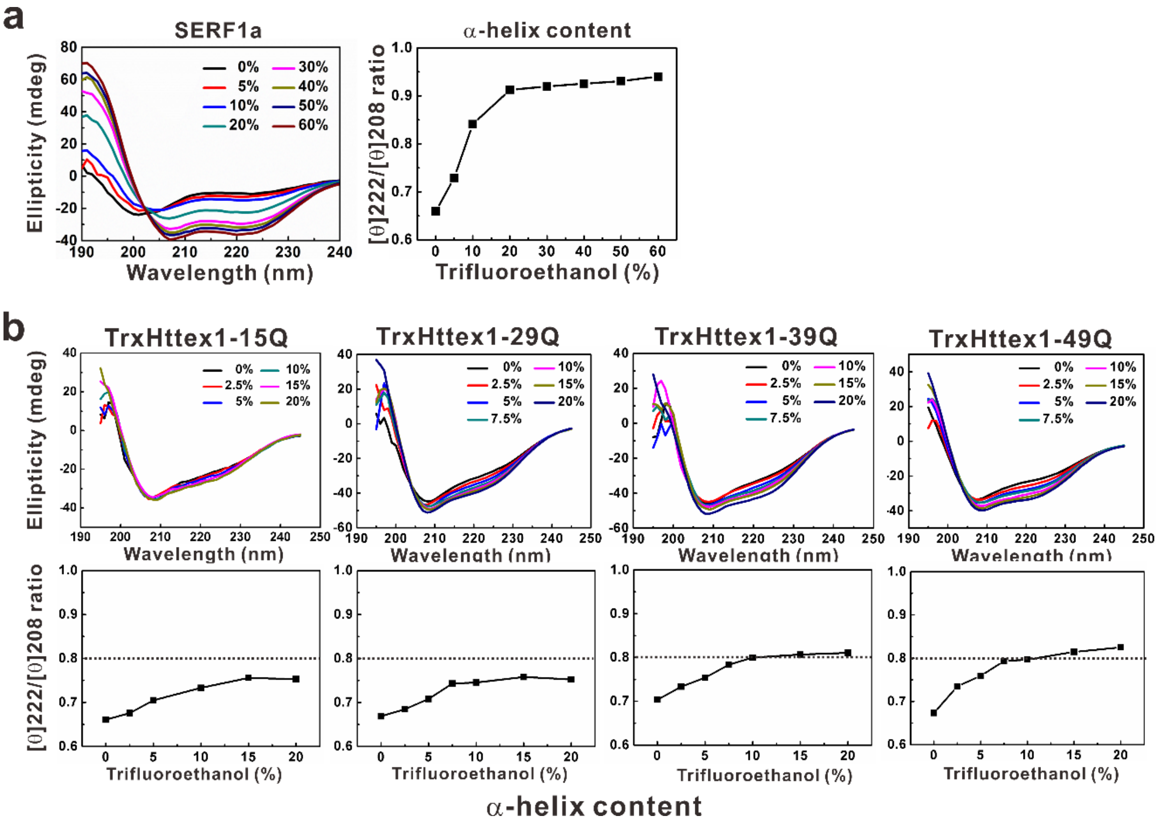

Supplementary Figure 6. Conformational transition of SERF1a and TrxHttex1-polyQ induced by TFE treatment. (a) Far-UV CD spectroscopy of SERF1a in various TFE concentrations (0%–60%). The ratio of ellipticity at 222 and 208 nm ([θ]222/[θ]208) was calculated and plotted against TFE concentration. **(b)** Far-UV CD spectroscopy ofTrxHttex1 proteins in various TFE concentrations. Fifty μM of TrxHttex1-15Q, TrxHttex1-29Q, TrxHttex1-39Q, and TrxHttex1-49Q were measured in 0%–20% TFE. The ratio of ellipticity at 222 and 208 nm ([θ]222/[θ]208) was calculated and plotted against TFE concentration.

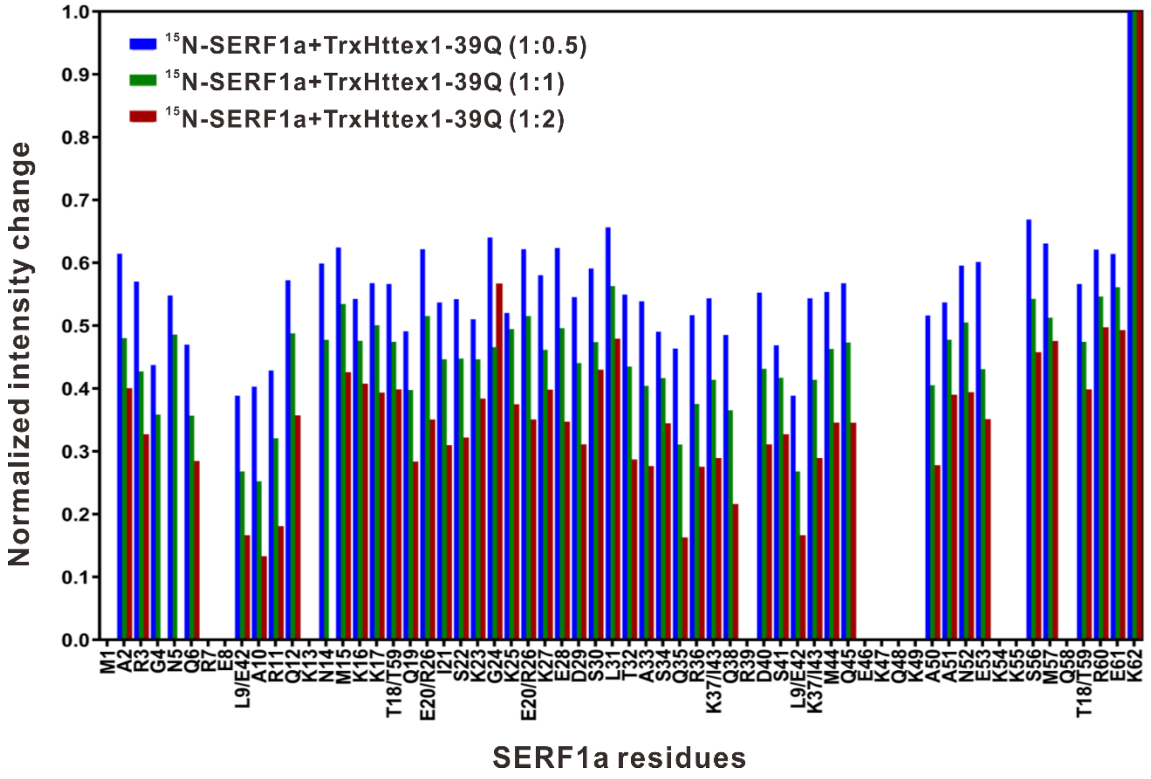

Supplementary Figure 7. NMR intensity drop between control and SERF1a with TrxHttex1-39Q (1:0.5, 1:1, and 1:2).

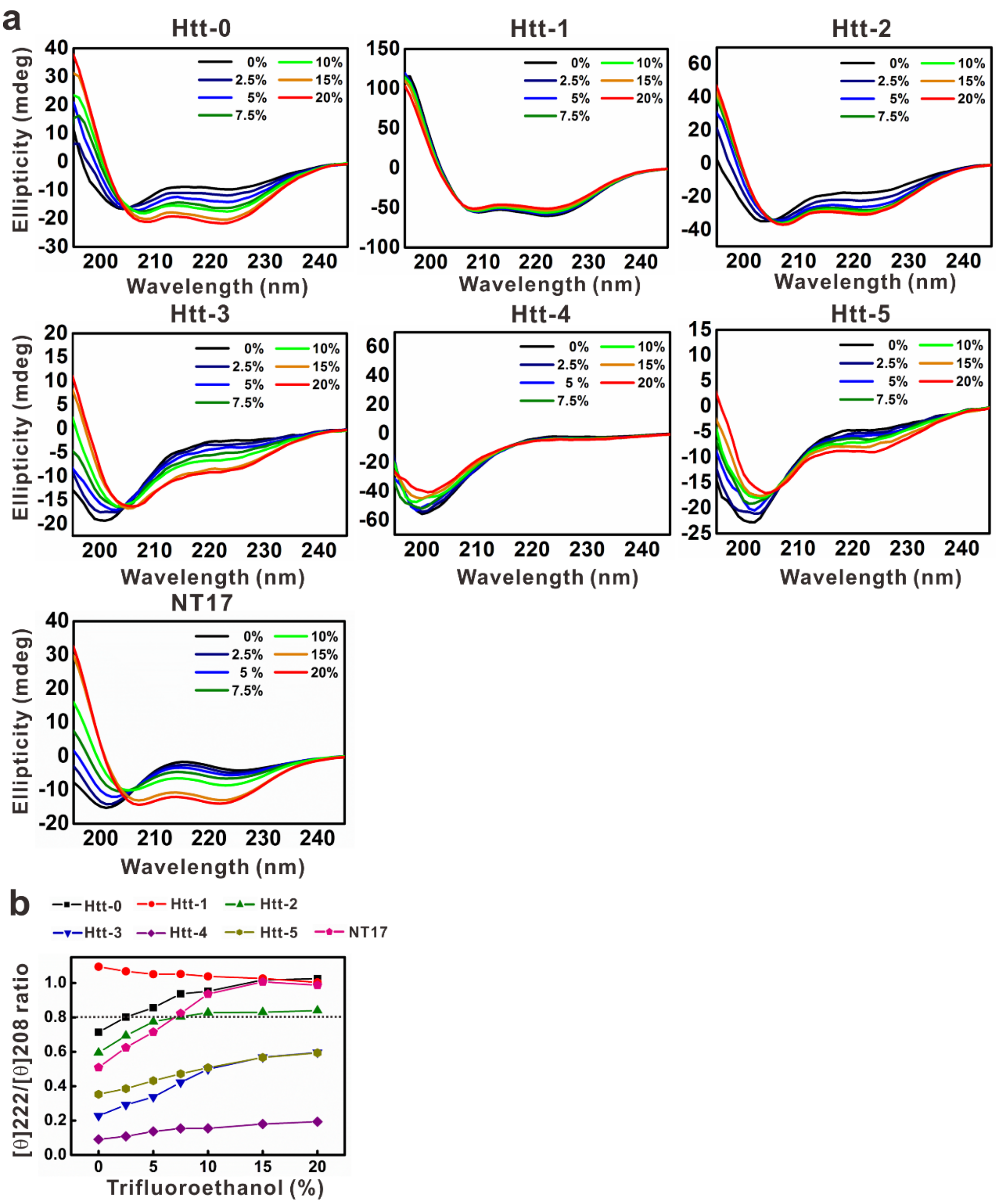

Supplementary Figure 8. Conformational changes in the synthesis of Htt peptides induced by TFE treatment. (a) Secondary conformation of Htt peptides, including 50 μM wild-type Htt-0, Htt-1, Htt-2, Htt-3, Htt-4, Htt-5, and NT-17, were detected by far-UV CD spectroscopy in the presence of 0%–20% TFE. **(b)** Ratio of ellipticity at 222 and 208 nm ([θ]222/[θ]208) plotted against TFE concentration.

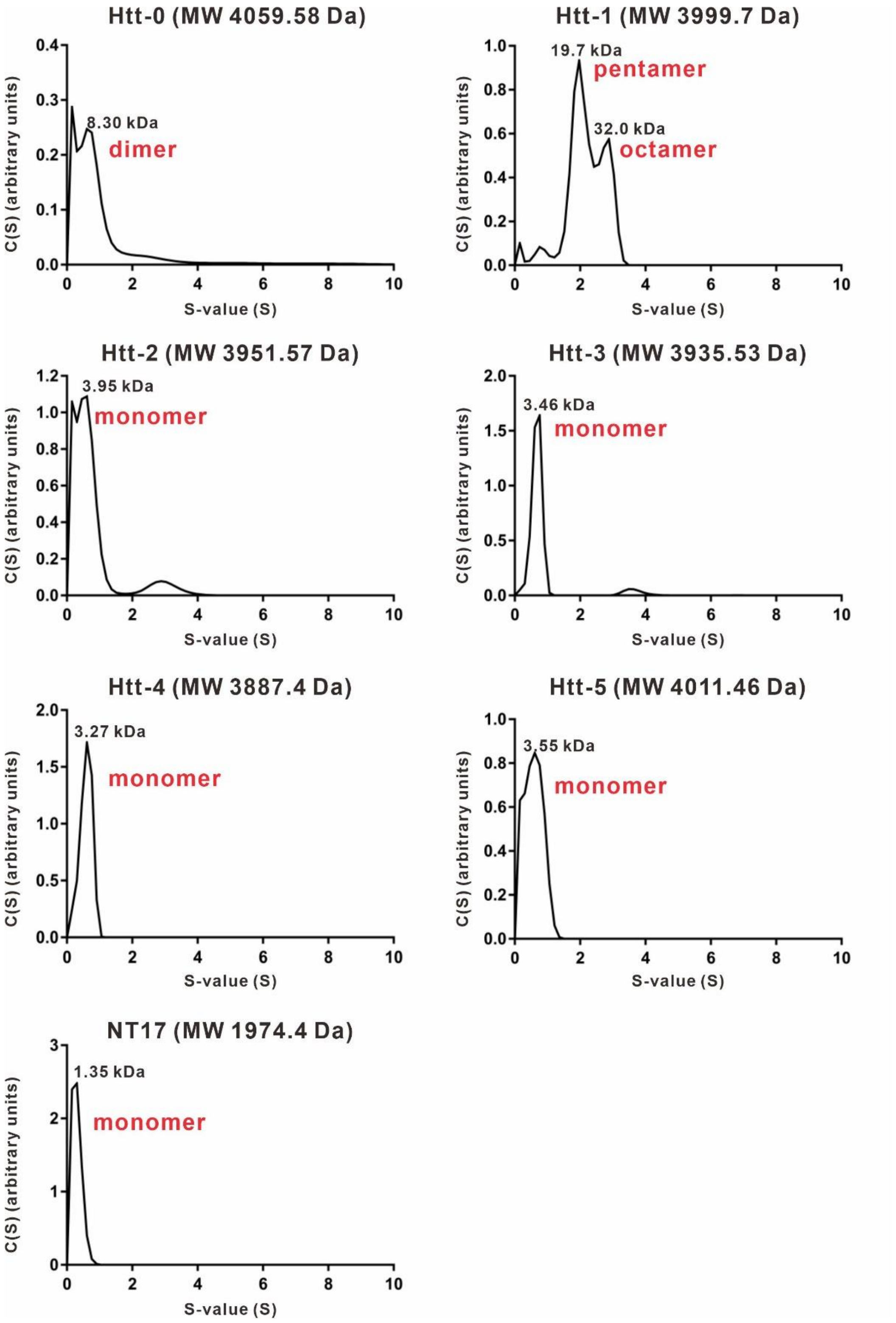

Supplementary Figure 9. AUC analysis of Htt peptides. NT17 and Htt-3 at 50 μM and Htt-0, Htt-1, Htt-2, Htt-4, and Htt-5 at 30 μM were centrifuged at 42,000 rpm for 24 h at 25 °C.

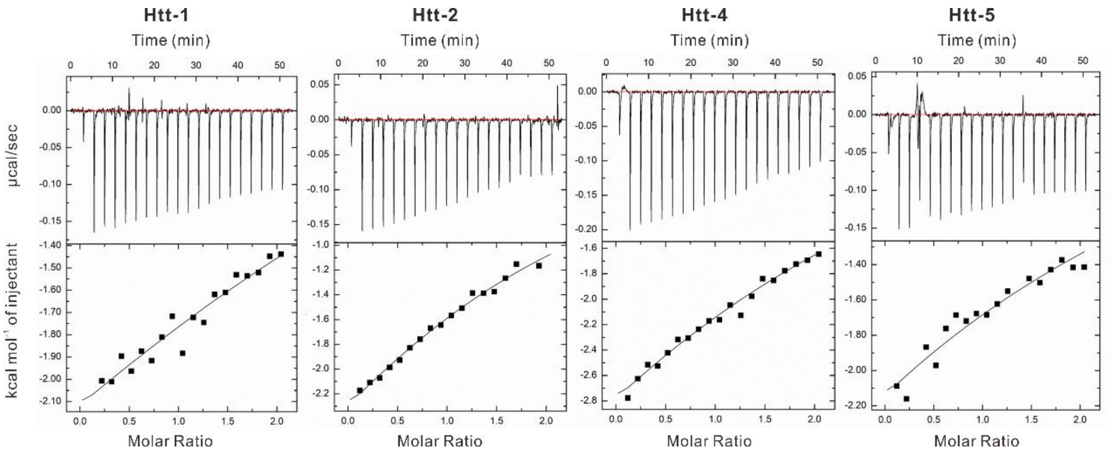

Supplementary Figure 10. Binding affinity of SERF1a and Htt peptides by isothermal titration calorimetry. ITC data of Htt-1, Htt-2, Htt-4, and Htt-5 were shown.

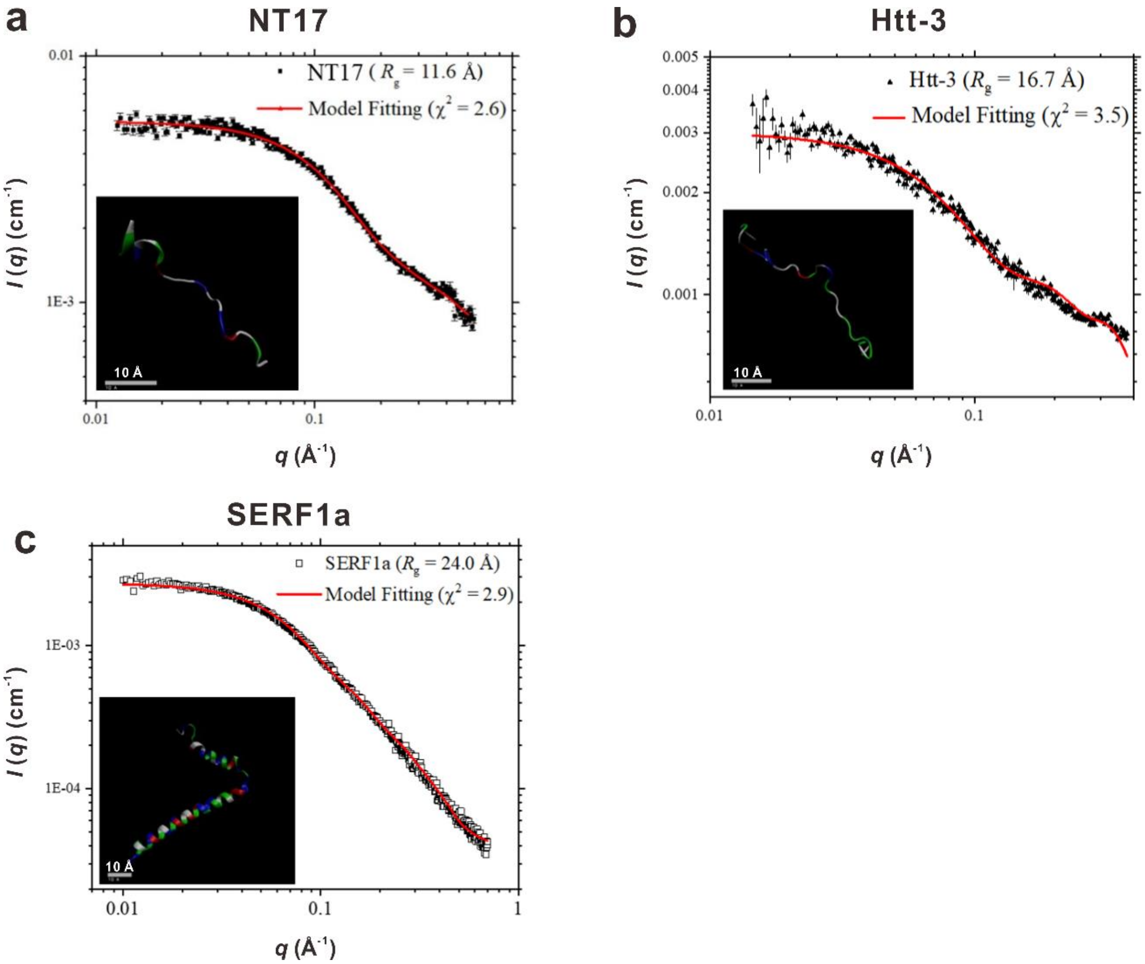

Supplementary Figure 11. SAXS data for **(a)** NT17 (1.0 mg/ml, 506.6 μM), **(b)** Htt-3 (1.5 mg/ml, 381.2 μM), and **(c)** SERF1a (4.1 mg/ml, 558.9 μM) fitted (solid curves) using the models shown. Scale bar, 10 Å.

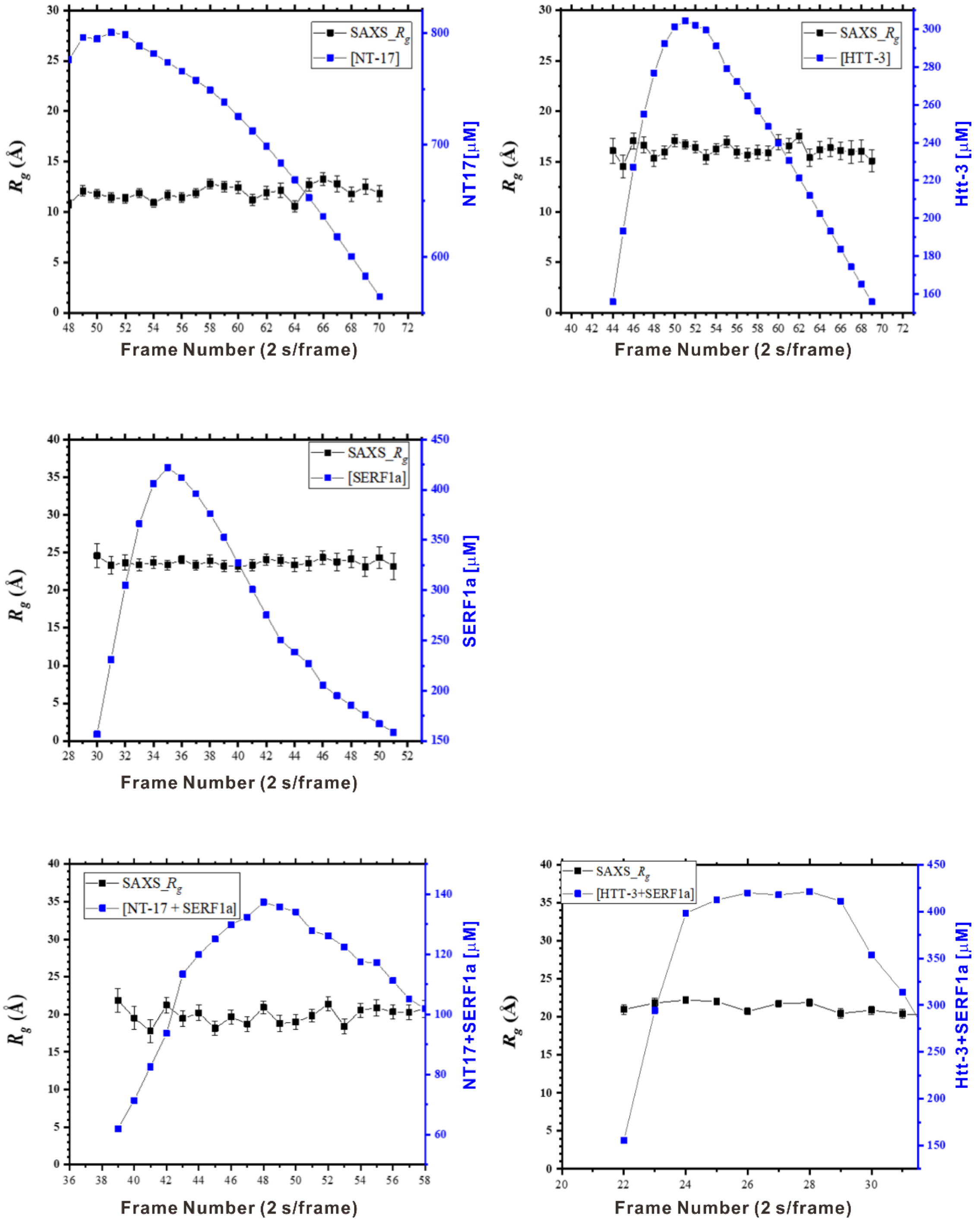

Supplementary Figure 12. The concentration independent R_g values data measured by SAXS for SERF1a, NT17, Htt-3, and the mixtures of SERF1a-NT17 and SERF1a-Htt-3. The error bars are the fitting uncertainties of the best-fitted *Rg* values from the corresponding frame of the SAXS data, using the Guinier approximation.

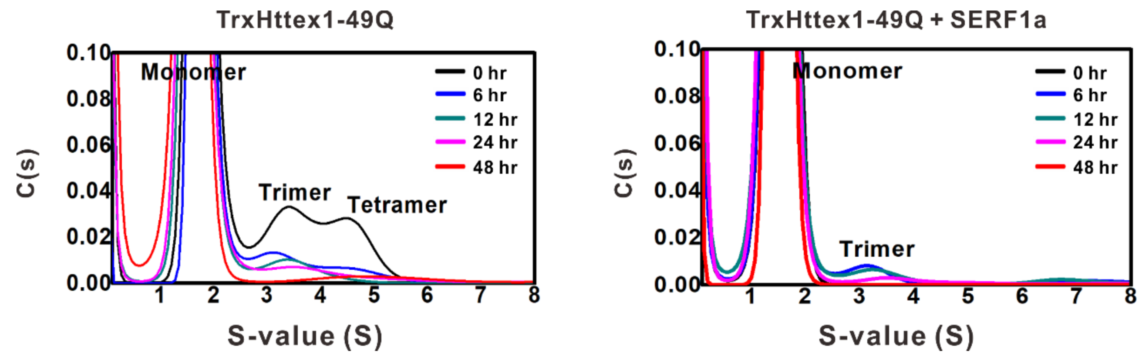

Supplementary Figure 13. Enlarged AUC analysis of TrxHttex1-49Q at different incubation times from Figure 5a.

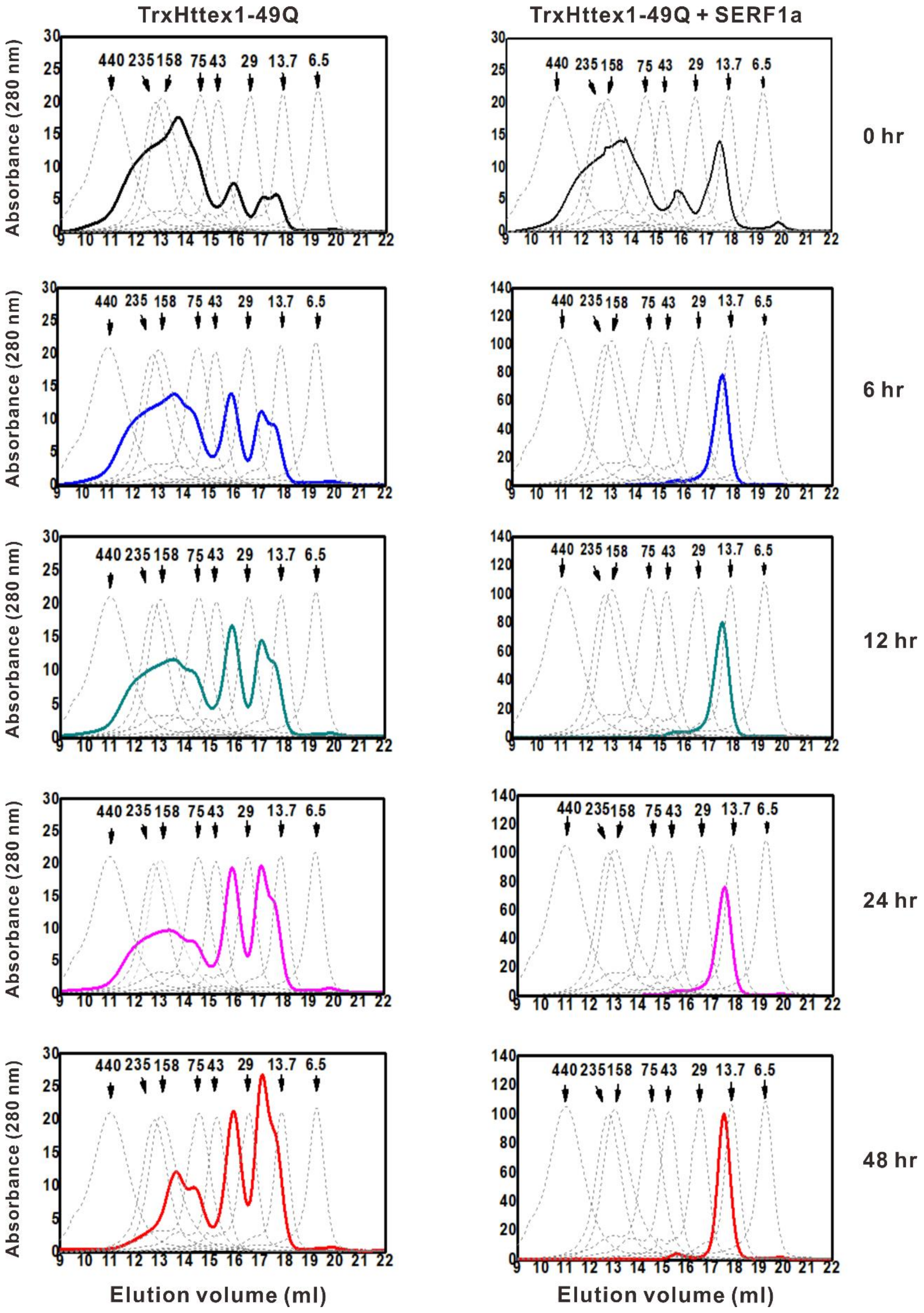

Supplementary Figure 14. Separated SEC chromatograms of TrxHttex1-49Q in the absence or presence of SERF1a at different incubation times with molecular weight controls.

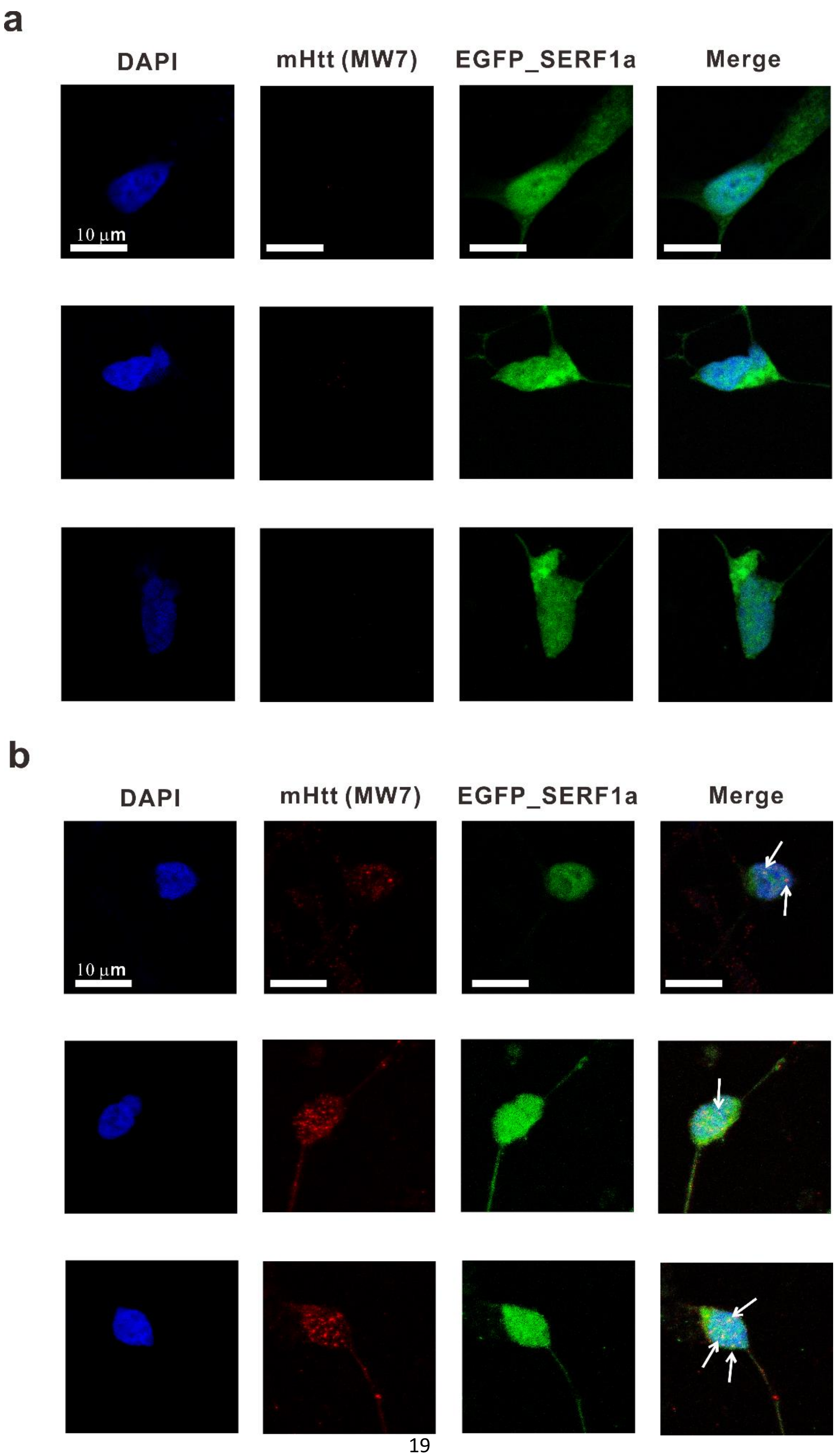
a
DAPI
mHtt (MW7)
EGFP_SERF1a
Merge
10 μm
b
DAPI
mHtt (MW7)
EGFP_SERF1a
Merge
10 μm

Supplementary Figure 15. Htt aggregates in control and HD iPSC-derived neurons. (a) Normal control strain C1 and **(b)** HD strain GM were infected by lentiviral constructs carrying EGFP control or EGFP_SERF1a. The mHtt aggregates were immune-stained by MW7 antibody, and the nucleus was stained by DAPI. The merged images of Htt (red), SERF1a (green), and nucleus (blue) were shown. Htt aggregates colocalized with SERF1a were indicated by white arrows. Scale bar, 10 μm.

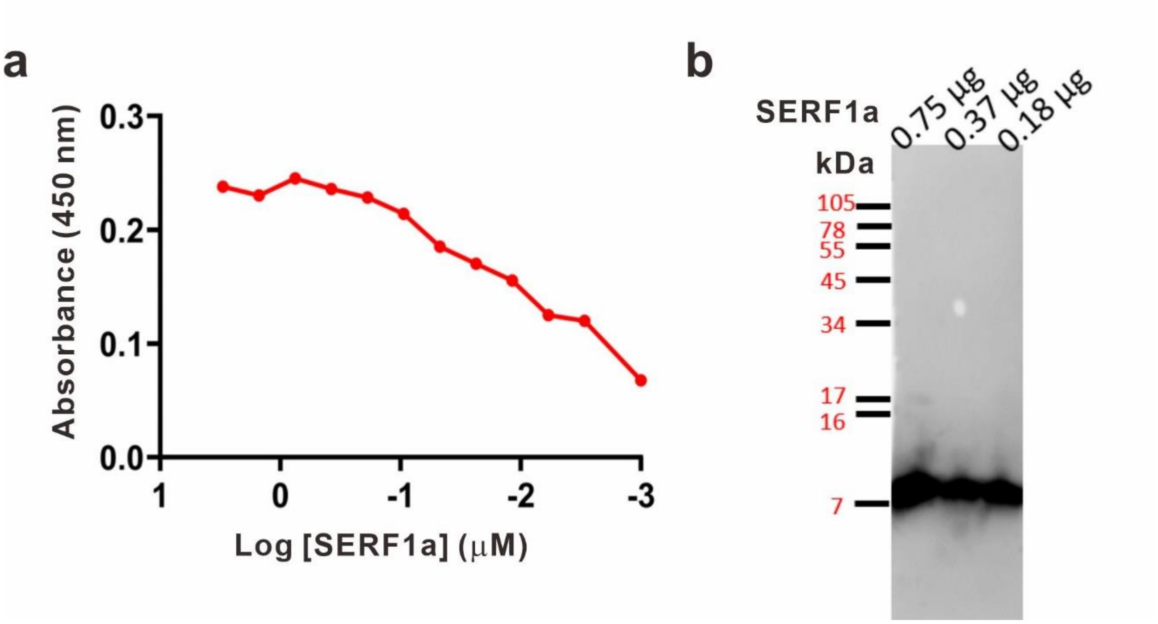

Supplementary Figure 16. Characterization of SERF1a antibody SERF#1 by ELISA and Western blot. (a) ELISA. Recombinant SERF1a was coated on the ELISA plate from 0 to 3 μM by serial dilution. SERF#1 antibody was added in 1:100 dilution. **(b)** Western blot. Recombinant SERF1a at 0.18, 0.37, 0.75 μg were loaded and subjected to Western blot probed by SERF#1 antibody.

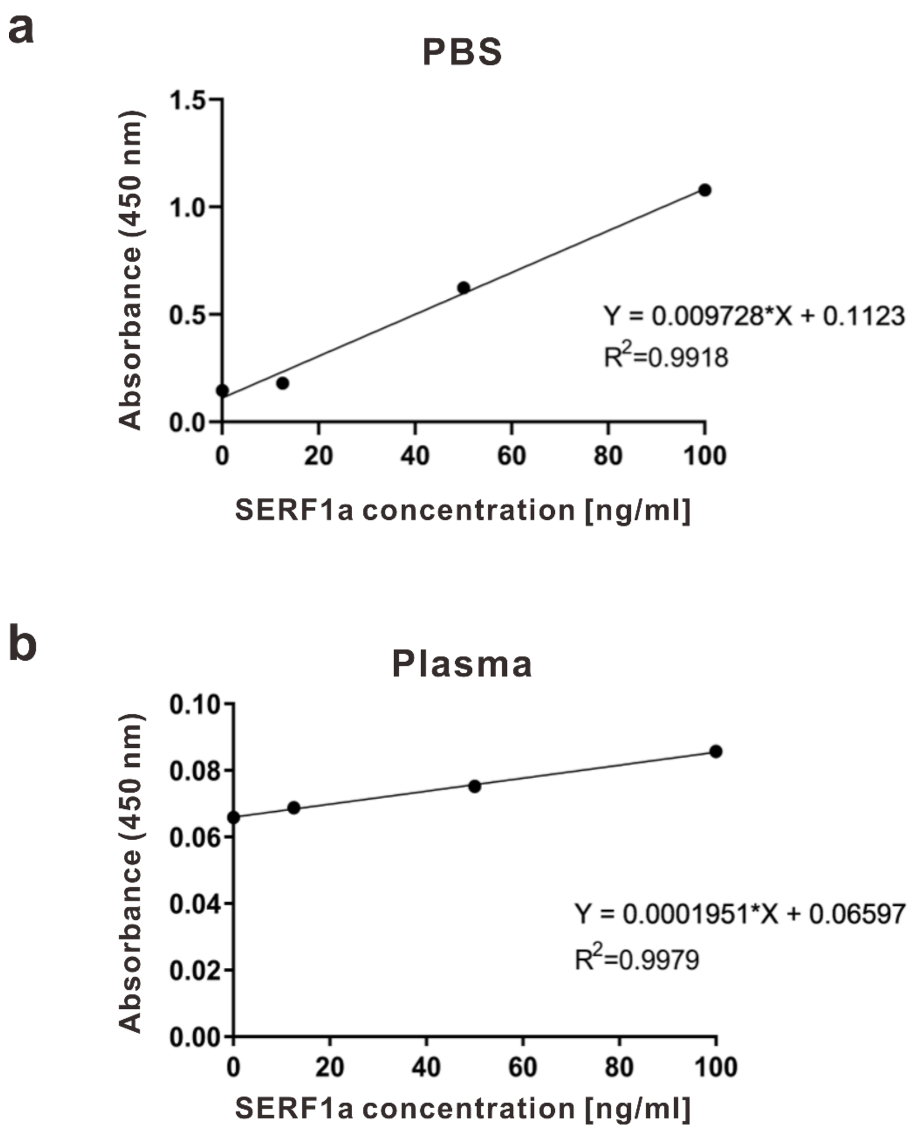

Supplementary Figure 17. Standard curves for calculating SERF1a concentration. (a) Recombinant SERF1a spike-in PBS. **(b)** Recombinant SERF1a spike-in normal plasma. The linear fitting equations and R^2 values were shown.

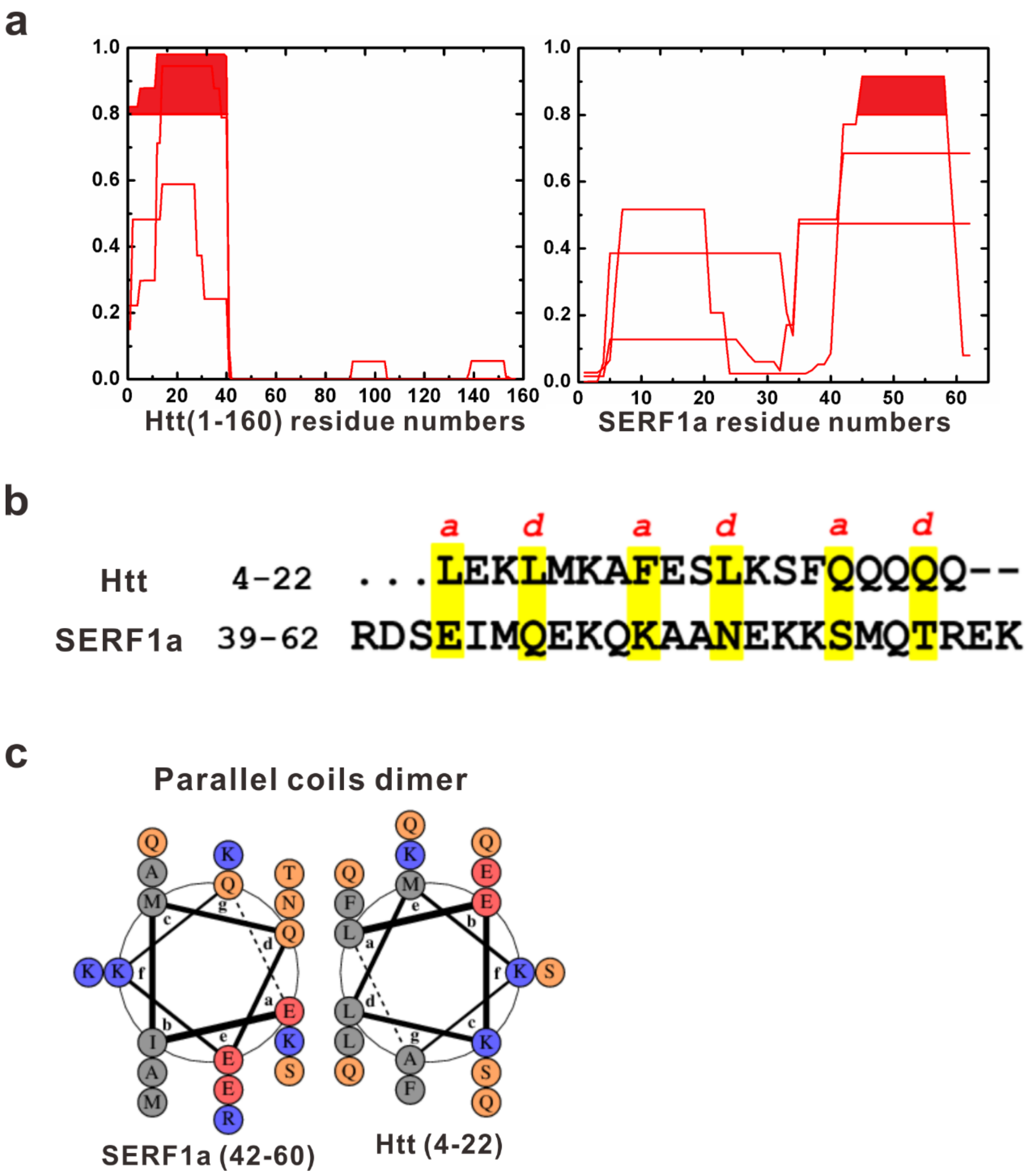

Supplementary Figure 18. Prediction for coiled-coils regions in SERF1a and Htt protein. (a) Per-residue coiled coil probability (0–1) for Htt (1–160) and SERF1a. Peaks with the highest probability (0.8–1) were highlighted. The N-terminus of Htt (residues 4–40) and the C-terminus of SERF1a (residues 39–62) had high probability of coiled coils. **(b)** Sequence alignment of the N-terminus of Htt and the C-terminus of SERF1a based on helical wheel prediction. Hydrophobic residues corresponding to those occupying a/d positions in the heptads were highlighted in yellow. **(c)** Possible coiled coil model of SERF1a/Htt heterodimer predicted using the program DrawCoil.

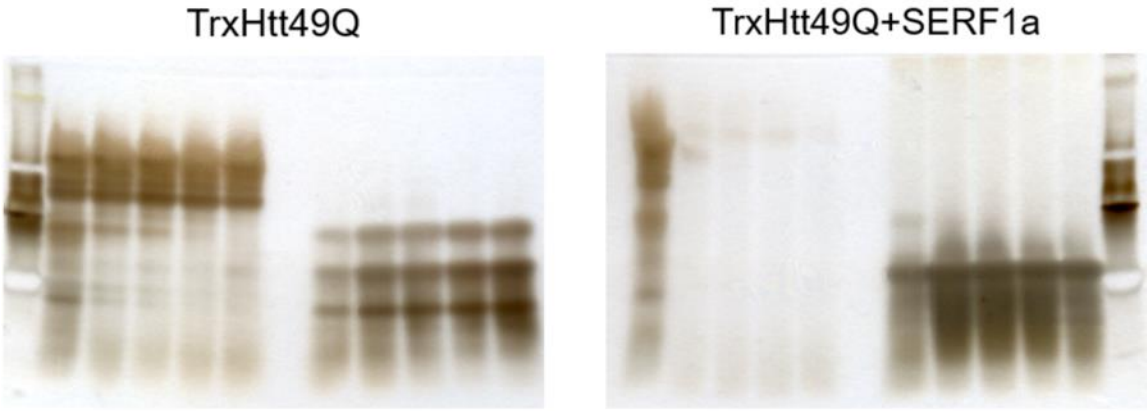

Supplementary Figure 19. Uncropped gel referring to Figure 5c

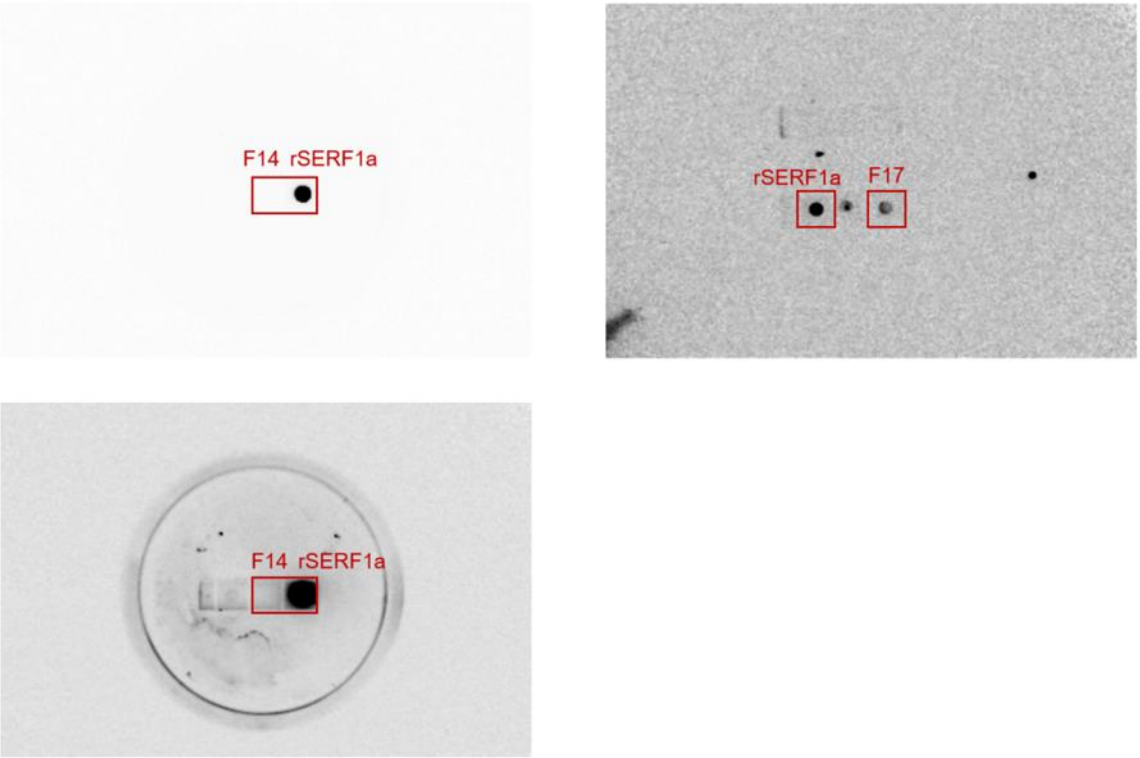

Supplementary Figure 20. Uncropped blots referring to Figure 5d

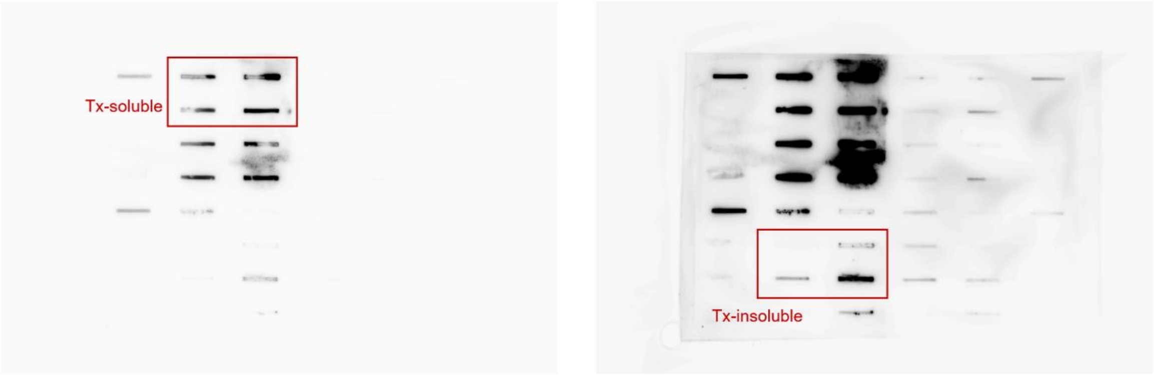

Supplementary Figure 21. Uncropped blots referring to Figure 6b

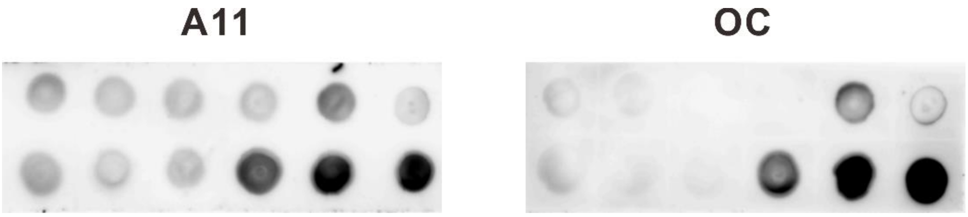

Supplementary Figure 22. Uncropped blots referring to Supplementary Fig. 3
