# Supplementary material for: Amyloid modifier SERF1a interacts with polyQ-expanded huntingtin-exon 1 via helical interactions and exacerbates polyQ-induced toxicity
Source: Commun Biol. 2023 Jul 21;6:767. doi: 10.1038/s42003-023-05142-0 (PMC10361993; doi:10.1038/s42003-023-05142-0)
Supplement: Supplementary file 5 — Reporting Summary [file 42003_2023_5142_MOESM5_ESM.pdf]

nature portfolio

Corresponding author(s): YRC

Last updated by author(s): Jun 30, 2023

Reporting Summary

Nature Portfolio wishes to improve the reproducibility of the work that we publish. This form provides structure for consistency and transparency in reporting. For further information on Nature Portfolio policies, see our Editorial Policies and the Editorial Policy Checklist.

Statistics

For all statistical analyses, confirm that the following items are present in the figure legend, table legend, main text, or Methods section.

n/a	Confirmed	
☐	☒	The exact sample size (n) for each experimental group/condition, given as a discrete number and unit of measurement
☐	☒	A statement on whether measurements were taken from distinct samples or whether the same sample was measured repeatedly
☐	☒	The statistical test(s) used AND whether they are one- or two-sided<br>*Only common tests should be described solely by name; describe more complex techniques in the Methods section.*
☒	☐	A description of all covariates tested
☐	☒	A description of any assumptions or corrections, such as tests of normality and adjustment for multiple comparisons
☐	☒	A full description of the statistical parameters including central tendency (e.g. means) or other basic estimates (e.g. regression coefficient) AND variation (e.g. standard deviation) or associated estimates of uncertainty (e.g. confidence intervals)
☒	☐	For null hypothesis testing, the test statistic (e.g. F, t, r) with confidence intervals, effect sizes, degrees of freedom and P value noted<br>*Give P values as exact values whenever suitable.*
☒	☐	For Bayesian analysis, information on the choice of priors and Markov chain Monte Carlo settings
☒	☐	For hierarchical and complex designs, identification of the appropriate level for tests and full reporting of outcomes
☒	☐	Estimates of effect sizes (e.g. Cohen's d, Pearson's r), indicating how they were calculated

Our web collection on statistics for biologists contains articles on many of the points above.

Software and code

Policy information about availability of computer code

Data collection	SpectraMax M5 (Molecular Devices) with SoftMax Pro 5.4<br>Hitachi H-7000 (Hitachi Inc., Tokyo, Japan) and FEI Tecnai G2 F20 S-TWIN transmission electron microscope with TEM user interface and DigitalMicrograph software<br>J-815 CD spectropolarimeter (Jasco Inc., Easton, MD, USA) with SpectraManager 2<br>FluoroMax-3 spectrofluorometer (Horiba Jobin Yvon, Kyoto, Japan) with FluorEssence V3.5<br>Bruker Avance 850 MHz NMR, Topspin2.1 (Bruker, Germany)<br>MicroCal iTC200 (GE)<br>Beckman Optima XL-I analytical ultracentrifugation (Beckman Coulter, USA)<br>Leica Automatic Upright Microscopy DM 6000B<br>Leica TCS-SP5-MP-SMD<br>ImageQuant LAS 4000
Data analysis	GraphPad Prism 9, Origin 9.0, Sparky version 3.114 (Goddard and Kneller), SEDNTERP (NIH), SEDFIT (U.S. NIH), Image J, MicroCal Analysis Launcher, ATSAS

For manuscripts utilizing custom algorithms or software that are central to the research but not yet described in published literature, software must be made available to editors and reviewers. We strongly encourage code deposition in a community repository (e.g. GitHub). See the Nature Portfolio guidelines for submitting code & software for further information.

Data

Policy information about availability of data

All manuscripts must include a data availability statement. This statement should provide the following information, where applicable:

- Accession codes, unique identifiers, or web links for publicly available datasets
- A description of any restrictions on data availability
- For clinical datasets or third party data, please ensure that the statement adheres to our policy

All data generated or analysed during this study are included in this published article (and its supplementary information files)

Human research participants

Policy information about studies involving human research participants and Sex and Gender in Research.

Reporting on sex and gender	Normal control: Male=9 Female=9<br>Patients: Male=13 Female=5
Population characteristics	Normal control: Male=9 average age: 43.3, sd: 13.0; Female=9 average age: 43, sd: 13.9<br>Patients: Male=13 average age: 41.5, sd: 13.4; Female=5 average age: 48.4, sd: 3.8
Recruitment	Human plasma samples from healthy individuals and HD patients were collected from Chang Gung Memorial Hospital at Linkou, Taiwan.
Ethics oversight	IRB in Chang Gung Memorial Hospital at Linkou and Academia Sinica (IRB01-12137)

Note that full information on the approval of the study protocol must also be provided in the manuscript.

Field-specific reporting

Please select the one below that is the best fit for your research. If you are not sure, read the appropriate sections before making your selection.

☒ Life sciences ☐ Behavioural & social sciences ☐ Ecological, evolutionary & environmental sciences

For a reference copy of the document with all sections, see nature.com/documents/nr-reporting-summary-flat.pdf

Life sciences study design

All studies must disclose on these points even when the disclosure is negative.

Sample size	Sample size is described in the manuscript and supplementary information
Data exclusions	Few concentration points in the intrinsic fluorescence experiments were excluded to get better fitting curve.
Replication	Replication is described in the manuscript and supplementary information
Randomization	Randomization was only applied for sample loading into the plates if possible
Blinding	Blinding was not possible and necessary for our study.

Reporting for specific materials, systems and methods

We require information from authors about some types of materials, experimental systems and methods used in many studies. Here, indicate whether each material, system or method listed is relevant to your study. If you are not sure if a list item applies to your research, read the appropriate section before selecting a response.

Materials & experimental systems

n/a	Involved in the study
☐	☒ Antibodies
☐	☒ Eukaryotic cell lines
☒	☐ Palaeontology and archaeology
☐	☒ Animals and other organisms
☒	☐ Clinical data
☒	☐ Dual use research of concern

Methods

n/a	Involved in the study
☒	☐ ChIP-seq
☒	☐ Flow cytometry
☒	☐ MRI-based neuroimaging

Antibodies

Antibodies used	1C2 (Millipore; MAB1574; 5TF1-1C2; 2038237), MW7 (Developmental Studies Hybridoma Bank; MW7), A11 (Invitrogen; AHB0052), OC (Millipore; AB2286), Anti-c-Myc (Sigma; M4439; 9E10), SERF#1 (in-house)
Validation	1C2 (mouse; ELISA, IC, IH(P), IP, WB), MW7 (mouse; ELISA, IF, IHC, WB), A11 (rabbit; WB, DB, ELISA, IHC), OC (rabbit; IP, IC, IH, ELISA, WB, DB), Anti-c-Myc (mouse; IP, immunoblotting, ELISA, IF, microarray, and electron microscopy), SERF#1 (mouse; IP, ELISA, WB, DB)

Eukaryotic cell lines

Policy information about cell lines and Sex and Gender in Research

Cell line source(s)	Neuro-2A (ATCC, Cat#CCL-131), HD-iPSC lines (GM), Human non-HD control iPSC lines (C1)
Authentication	No further authentication applied in addition to the authentication by ATCC
Mycoplasma contamination	No further tests applied in addition to the tests by ATCC
Commonly misidentified lines (See ICLAC register)	N/A

Animals and other research organisms

Policy information about studies involving animals; ARRIVE guidelines recommended for reporting animal research, and Sex and Gender in Research

Laboratory animals	HD transgenic mice (R6/2) and wild type littermate
Wild animals	The study did not involve wild animals
Reporting on sex	Sex was not considered in our study.
Field-collected samples	The study did not involve samples collected from the field
Ethics oversight	Academia Sinica IACUC

Note that full information on the approval of the study protocol must also be provided in the manuscript.
